# Appendiceal mucocele masquerading as an epithelial borderline ovarian tumor: A case report from Somalia

**DOI:** 10.1016/j.ijscr.2024.110658

**Published:** 2024-11-24

**Authors:** Nuur Mucaawiye Muse, Abdirahman Ibrahim Said, Ismail Mohamed, Abdilahi Hussein, Mohamed Omer Hussein, Hassan Sh. Abdirahman Elmi

**Affiliations:** a College of Health Science, School of Medicine, Amoud University, Borama, Somalia; b Department of Biology, Amoud University, Borama, Somalia; c Department of Surgery, Borama Regional Hospital, Borama, Somalia; d Faculty of Science, Charles University, Prague, Czech Republic

**Keywords:** Appendiceal mucocele, Diagnosis, Surgical resection, Complications, Multidisciplinary

## Abstract

**Introduction:**

Appendiceal mucocele, a rare condition characterized by mucoid material accumulation in the appendix, often presents asymptomatically. Diagnosis can be challenging, and surgical resection is crucial to prevent complications. We report a case managed in a low-resource setting, highlighting the importance of early identification.

**Case presentation:**

A 56-year-old postmenopausal woman presented with right lower quadrant pain and a pelvic mass. Imaging revealed a cystic lesion and exploratory laparotomy was performed. Intraoperatively, a mucocele of the appendix was discovered. An appendectomy was performed, and a histopathological examination confirmed a serous borderline tumor, this work has been reported in line with the SCARE criteria.

**Discussion:**

Preoperative diagnosis of appendiceal mucoceles is difficult due to their clinical variability. Surgical intervention is essential, with meticulous resection required to prevent peritoneal contamination. The absence of intraoperative pathology consultation in this case underscores the need for greater access to specialized resources, including pathology services, in resource-limited settings to ensure accurate diagnosis and appropriate surgical management.

**Conclusion:**

This case highlights the critical importance of clinical suspicion for appendiceal mucoceles, even when initial radiological findings are inconclusive. The lack of intraoperative pathology consultation underscores the need for specialized pathology services in resource-limited settings. Given the increased risk of colonic adenocarcinoma, multidisciplinary collaboration and diligent post-operative surveillance are crucial for optimizing patient outcomes.

## Highlights

• An appendiceal mucocele found during ovarian surgery
• The lack of intraoperative pathology consultation highlights resource gaps.
• Increased awareness and timely intervention can prevent serious complications.

## 1 Introduction

Appendiceal mucocele is characterized by an obstructive dilation of the appendix caused by the accumulation of mucoid material within the lumen. It is a rare condition, with an incidence ranging from 0.2 % to 0.7 % of all appendectomy specimens [1]. Diagnosis of appendiceal mucocele is often challenging due to the frequently asymptomatic presentation. In approximately 50 % of cases, the lesion is discovered incidentally during surgery, such as appendectomy or other abdominal procedures [2]. Careful surgical resection is crucial to avoid perforation, which could result in the dissemination of malignant cells (e.g., cystadenocarcinoma) into the peritoneal cavity, significantly affecting the prognosis [2]. Additionally, the removal of the mucocele is essential to prevent rupture, which, in cases of neoplastic lesions, may lead to pseudomyxoma peritonei, a serious condition associated with poor outcomes [3]. We present a case managed in a low-resource setting, where the diagnosis was made intraoperatively by the surgeon during a procedure for a patient under the care of the obstetrics department. This highlights the importance of clinicians considering appendiceal mucocele in the differential diagnosis, as early identification can prevent complications.

## 2 Case presentation

A 56-year-old postmenopausal woman was admitted to the gynecology department for a symptomatic pelvic mass associated with RLQ pain for six months. She had a medical history of hypertension and diabetic. Her personal surgical and familial histories were unremarkable. Physical examination revealed a mild -tender, palpable mass in the right lower quadrant. The results of the other abdominal examinations were unremarkable. A gynecological examination showed a normal uterus and cervix.

**Figs. 1 & 2:**
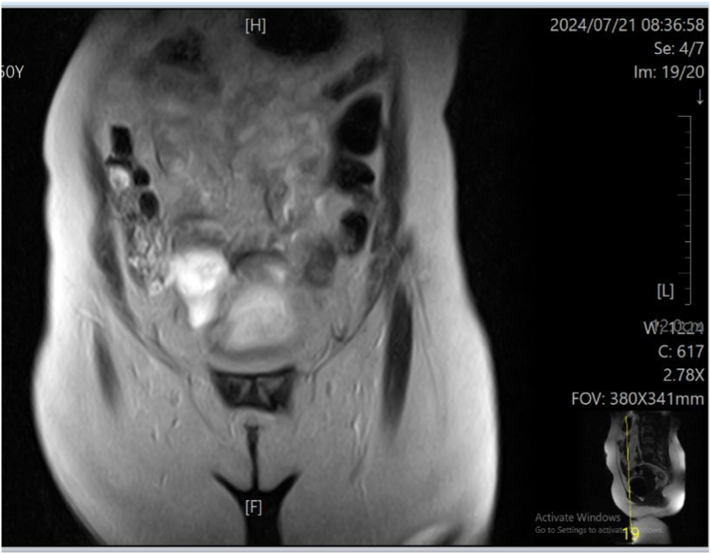

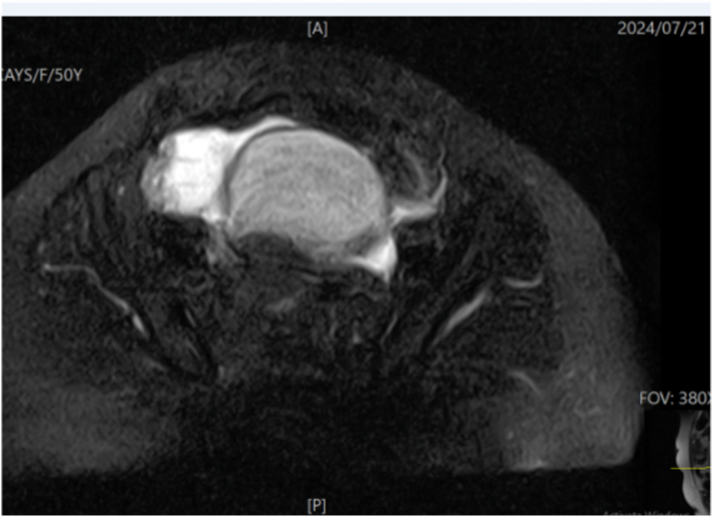
MRI findings demonstrating a benign-appearing pelvic lesion on the right ovary, most likely a cystadenoma, accompanied by pelvic inflammatory disease with significant localized fluid collection in the pouch of Douglas.

Initial laboratory evaluation revealed elevated white blood cell count (leukocytosis, 13 ∗ 10^9^/L). All other laboratory parameters, including chemistry panels, coagulation panels, and urine analysis, were within normal limits. The Transabdominal ultrasound showed a normal uterus, with a right ovarian mass of 6.14 cm ∗ 4.7 cm. The left adnexa were normal in the ultrasound. Pelvic MRI revealed a well-defined, smooth, ovoid, right ovarian lesion measuring 47 mm × 54 × 52 mm. The lesion demonstrated mixed intensity, appearing partially solid and partially cystic, see (Fig. 1).

The gynecologist performed an explorative laparotomy, and the intraoperative findings included a cystic mass in the right pelvic area which originated from the appendix. The uterus both fallopian tubes and ovaries were normal. The other specific findings were unremarkable. The general surgeon was called to confirm that the mass originated from the appendix. Both the cystic portion and the base of the appendix were enlarged to similar diameters similar to that of the cecum. Serosal invasion, regional lymph node enlargement, and ascites were not observed (Fig. 2).

There were no suspicious findings of the peritoneum and liver. Based on these findings, an appendectomy was performed.

**Fig. 3:**
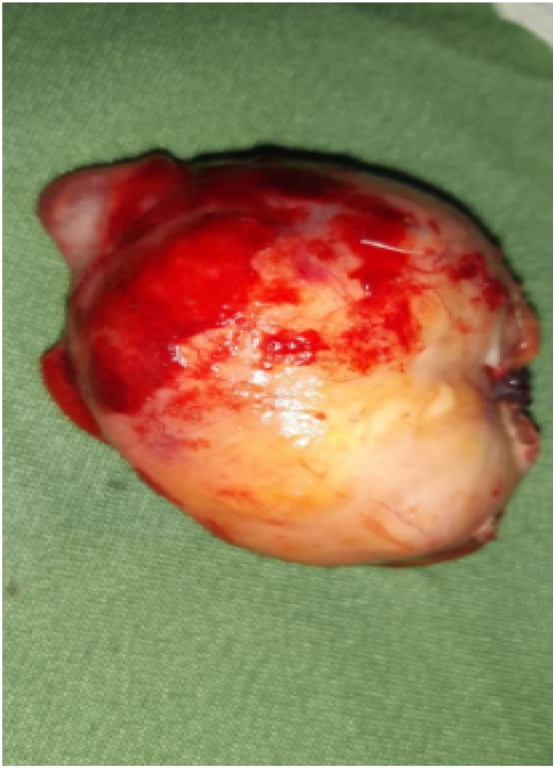
Gross appearance of cystic appendicular mass post-surgery.

Gross examination revealed an enlarged appendix measuring 10 × 6.0 × 5.5 cm. The distal portion was whitish and had a huge cystic feature, while the proximal portion had a serosal appearance and was enlarged. The lumen was filled with mucoid material and was perforated (Fig. 3).

Histopathological examination confirmed a serous borderline tumor originating from the appendix. The resection margin was clear. The patient was discharged on the fifth postoperative day without any complications. The outpatient follow-up performed approximately 1 month later showed no evidence of disease progression or any other symptoms. This work has been reported in line with the SCARE criteria [4].

## 3 Discussion

The mucocele of the appendix, first described by Rokitansky in 1842, is characterized by cystic dilation of the appendix due to the accumulation of mucus secretion, and it can be classified based on the histopathological features of lumen obstruction. A simple mucocele, also known as an inflammatory, obstructive, or retention cyst, results from degenerative changes in the epithelium, leading to obstruction and subsequent distension of the appendix. In contrast, hyperplastic mucocele occurs due to hyperplastic growth of the appendiceal or cecal mucosa, like hyperplastic polyps found in the colon. Mucinous cystadenoma represents a neoplastic process with dysplastic epithelium akin to adenomatous polyps of the colon, while mucinous cystadenocarcinoma is a more aggressive variant that exhibits high-grade cellular dysplasia, stromal invasion, and extension beyond the muscularis mucosae, distinguishing it as a malignant entity [5].

The preoperative diagnosis of mucoceles and appendiceal tumors remains difficult due to their clinical variability. These tumors may be asymptomatic and discovered incidentally, or they may present symptoms that mimic acute appendicitis. In some cases, patients may experience vague symptoms such as chronic abdominal pain, urinary retention, constipation, or, in later stages, new-onset umbilical or inguinal hernias caused by increased intra-abdominal pressure [6]. Radiological imaging is essential in diagnosis, with contrast-enhanced CT scans considered the gold standard, providing 95 % sensitivity in detecting appendiceal tumors in symptomatic patients. MRI is preferred for staging and follow-up, as it offers higher sensitivity and specificity than CT, particularly in identifying peritoneal spread. Despite these advances, imaging is not infallible, as evidenced by cases where tumors are misdiagnosed or confused with other conditions, such as ovarian masses [6].

However, one of the key factors in improving diagnostic accuracy is for clinicians to maintain a high index of suspicion for these tumors, particularly when patients fit the typical profile. Appendiceal mucoceles are mostly found in adults between the ages of 50 and 60 years, and studies indicate a higher prevalence among females, with a sex ratio of approximately four females to one male. In this case, the patient-matched the common demographic profile, underscoring the importance of considering appendiceal mucoceles as part of the differential diagnosis when clinical and radiological findings are ambiguous [7].

Mucoceles of the appendix are typically managed through surgical intervention, with preoperative diagnosis playing a critical role in guiding the meticulous mobilization and resection needed to prevent peritoneal contamination. In cases where malignancy is suspected—either based on preoperative imaging or intraoperative frozen section analysis—a right hemicolectomy is often recommended. Furthermore, given that patients with appendiceal mucocele have a sixfold increased risk of developing colonic adenocarcinoma compared to the general population, routine colonic surveillance is advised to monitor and mitigate this elevated risk [8].

It is important to highlight the critical role of multidisciplinary management and intraoperative consultation in surgical practice, particularly in gynecologic surgery, where intraoperative consultation is frequently employed to assist the surgeon in determining the appropriate extent of surgery [9]. In this case, the surgical team was adequately prepared to manage the patient, despite being in a setting where multidisciplinary teams are often unavailable, and healthcare professionals are overburdened. This underscores the importance of multidisciplinary collaboration to ensure comprehensive patient care.

However, for this patient, intraoperative pathology consultation—which is crucial in surgeries of this nature for assessing the extent of cancer spread [9]—was not available. As a result, the definitive diagnosis was delayed and only confirmed four days later, highlighting the significant challenges and limitations faced in resource-limited settings.

## 4 Conclusion

Appendiceal mucoceles present diagnostic and management challenges, especially in resource-limited settings. Their varied symptoms can delay diagnosis, making radiological imaging and clinical suspicion essential. Surgical intervention, often needed, requires careful planning to avoid complications. The lack of intraoperative pathology highlights the value of multidisciplinary support for accurate diagnosis. Due to the elevated risk of associated colonic adenocarcinoma, close postoperative monitoring is crucial. This case underscores the importance of comprehensive, multidisciplinary approaches in challenging healthcare environments to improve patient outcomes.

## Ethical approval

Ethical approval for this study was obtained.

## Consent for publication

A written informed consent was obtained from the patient for publication and any accompanying images.

## Guarantor

Dr. Hassan sh Abdirahman Elmi.

## Funding

This research did not receive any financial support from any external sources.

## Author contribution

All the authors in this work participated in the patient caring and treatment, conceptualization of the case report, drafted the manuscript, and reviewed the final version of the case report.

## Conflict of interest statement

The authors affirm that there are no conflicts of interest in this article's publication.
